# The effect of mindfulness training on resting-state networks in pre-adolescent children with sub-clinical anxiety related attention impairments

**DOI:** 10.1007/s11682-022-00673-2

**Published:** 2022-05-18

**Authors:** Michelle Kennedy, Abdalla Z. Mohamed, Paul Schwenn, Denise Beaudequin, Zack Shan, Daniel F. Hermens, Jim Lagopoulos

**Affiliations:** Thompson Institute, University of the Sunshine Coast, 12 Innovation Parkway, Birtinya, Queensland 4575 Australia

**Keywords:** fMRI, anxiety, attention, mindfulness, children

## Abstract

Mindfulness training has been associated with improved attention and affect regulation in preadolescent children with anxiety related attention impairments, however little is known about the underlying neurobiology. This study sought to investigate the impact of mindfulness training on functional connectivity of attention and limbic brain networks in pre-adolescents. A total of 47 children with anxiety and/or attention issues (aged 9-11 years) participated in a 10-week mindfulness intervention. Anxiety and attention measures and resting-state fMRI were completed at pre- and post-intervention. Sustained attention was measured using the Conners Continuous Performance Test, while the anxiety levels were measured using the Spence Children’s Anxiety Scale. Functional networks were estimated using independent-component analysis, and voxel-based analysis was used to determine the difference between the time-points to identify the effect of the intervention on the functional connectivity. There was a significant decrease in anxiety symptoms and improvement in attention scores following the intervention. From a network perspective, the results showed increased functional connectivity post intervention in the salience and fronto-parietal networks as well as the medial-inferior temporal component of the default mode network. Positive correlations were identified in the fronto-parietal network with Hit Response Time and the Spence Children’s Anxiety Scale total and between the default mode network and Hit Response Time. A 10-week mindfulness intervention in children was associated with a reduction in anxiety related attention impairments, which corresponded with concomitant changes in functional connectivity.

## Introduction

Attention, involving the concentration of one’s awareness on a given task to the exclusion of distracting stimuli, is often compromised in anxiety states (Petersen & Posner, 2012). Specifically, emotion regulation requires cognitive control to minimize attention shifts to irrelevant stimuli, as dysfunction in these processes can produce deficits in inhibitory control (Eysenck et al., 2007). Cognitive control and emotion regulation processes develop throughout childhood, and disruption to maturing brain networks during this period may contribute to dysfunction in these processes (Davidson et al., 2006; Luna et al., 2004). The networks associated with these processes involve the Salience Network (SLN), Fronto-Parietal Network (FPN) and Default Mode network (DMN) referred to as the resting state networks (RSNs) (Petersen & Posner, 2012). Child studies investigating changes in RSNs functional connectivity (FC) are limited (de Bie et al., 2012; Jolles et al., 2011; Muetzel et al., 2016), with evidence demonstrating a protracted development from short-range connections in childhood to long-distance connections in adulthood (Betzel et al., 2014; Fair et al., 2009; Thomason et al., 2011; Uddin et al., 2010). These developmental differences suggest functional networks may be less specialized or efficient during childhood, thus may underlie atypical cognitive development and deficits in emotion regulation, commonly associated with anxiety and attention impairments (Jolles et al., 2011; Supekar et al., 2009).

Mindfulness interventions have been associated with altered functional connectivity (FC) (Brefczynski-Lewis et al., 2007; Jha et al., 2007) in resting-state networks (V. Menon, 2015; Sha et al., 2019) and have been shown to improve attention and symptoms of anxiety in adult populations (Hofmann et al., 2010)*.* Changes in FC in these networks can be measured at rest using resting-state functional magnetic resonance imaging (rsfMRI) (Raichle et al., 2001; Tang et al., 2015).

Mindfulness studies investigating FC in resting-state networks in children are limited (Bauer et al., 2020; Marusak et al., 2018), with most focusing on clinical disorders or the efficacy of school-based mindfulness programs (Dunning et al., 2019; Flook et al., 2010). There is limited neurobiologically-informed research investigating the efficacy of mindfulness training on subclinical anxiety-related attention impairments in children.

The current study aimed to investigate the effect of mindfulness training on anxiety-related attention impairments and FC following a 10-week mindfulness intervention in preadolescent children. We hypothesized that mindfulness training would decrease anxiety symptoms and improve attention issues and would be associated with changes in FC within the networks that sub-serve these domains.

## Methods

### Participants

Children aged 9 – 11 years of age, experiencing symptoms of anxiety and/or inattention, and proficient in spoken and written English were included in the study. Children with formal diagnoses of anxiety, attention, or autism spectrum disorder were excluded from the study. Children with neurological disorders (e.g. epilepsy), intellectual disability, major medical illness, or who had sustained head injury (with loss of consciousness exceeding 10 minutes) were also excluded. Eligibility for the study was defined by parent-report of no disorder diagnosis and not taking any psychotropic medication during a telephone interview. The children were recruited through the University of Sunshine Coast’s social media platform and advertisements in various school newsletters. Individual children in years 4-6 (not class groups), from a variety of state and private schools across the Sunshine Coast participated in the pre- and post- assessments and intervention, which were conducted at the Thompson Institute. The one hour intervention was offered three afternoons per week, two sessions per afternoon, with participants selecting one session to attend per week. The group size ranged from 8-12 participants and groups were mixed ages. Of *N=82* expressions of interest received, *N=49* met inclusion criteria. Two children withdrew during the study, thus *N=47* participants completed the intervention and pre- and post-intervention assessments.

### Procedures

Participants undertook the following assessments with a total duration of 60 minutes: (i) the Spence Children’s Anxiety Scale (SCAS), (ii) the Conners Continuous Performance Test 3rd edition (CPT 3) and (iii) magnetic resonance imaging (MRI) scanning. Assessments were conducted at two time-points: (i) pre-intervention (‘pre’); up to two weeks before the 10-week mindfulness intervention; and (ii) post-intervention (‘post’); up to two weeks following the mindfulness intervention. The study was conducted at the Thompson Institute, University of the Sunshine Coast, between July 2019 and January 2020.

#### The Spence Children’s Anxiety Scale (SCAS)

The SCAS assessment (Spence, 1998) was used to provide an overall self-report measure of anxiety symptoms, as well as six childhood anxiety disorder subscale scores (separation anxiety, generalized anxiety, social phobia, panic/agoraphobia, obsessive-compulsive disorder and fear of physical injuries) in accordance with DSM-5 criteria. The participants rated 38 statements relating to their fears, worries, and somatic symptoms using a Likert scale ranging from 0 (*never*) to 3 (*always*), with a possible score ranging from 0 - 114. There is empirical support for test-retest reliability and internal consistency across child and adolescent populations (Spence et al., 2003). The SCAS demonstrates good construct validity, and when compared with the Strength and Difficulty Questionnaire supported the SCAS’s convergent and divergent validity (Arendt et al., 2014; Spence et al., 2003).

#### The Conners Continuous Performance Test 3^rd^ edition (CPT 3)

The CPT 3 is a computerized test of attention and attention related problems in children 8-18 years (Conners et al., 2003). The CPT 3 measured four aspects of attention including sustained attention, inattentiveness, impulsivity and vigilance, via 360 trials presented in 14 minutes. Variables measured included detectability, errors of commissions, omissions and hit response time.

#### Mindfulness intervention

The Mindfulness in Schools Project .b course is a school-based mindfulness curriculum, selected for demonstrated improvements in psychological well-being and cognitive skills post-intervention (Kuyken et al., 2013; Wilde et al., 2019). The mindfulness intervention included mindfulness strategies that were non-directive (encouragement of allowing thoughts to come and go) and attention focused (focusing attention on the breath). Mindfulness skills were developed through concepts that included paying attention, recognising worry, letting go of thoughts, dealing with difficult emotions and gratitude. The program was delivered face to face by a trained program facilitator with supporting animations. Participants were required to practice the mindfulness skills taught each week at home at least 3-4 times per week, supported by access to a parent hub that explained to parents the type of skill to be practiced.

#### Magnetic resonance imaging

Imaging was performed on a Siemens Skyra 3T scanner (Siemens, Erlangen) using a 64-channel head and neck coil at the Nola Thompson Centre of Advanced Imaging, Thompson Institute. The T1-weighted images were collected using magnetization prepared-rapid gradient echo sequence with TR/TE/flip angle =2000ms/1.7ms/7^o^; matrix = 224 x 224 x 176, and resolution = 1.027 x 1.027 x 1 mm. The rsfMRI was acquired using EPI with TR/TE = 1600ms/30ms, matrix=74 x 74 x 48, resolution of 3.027 x 3.027 x 3.0 mm, and a total of 300 volumes. The resting state fMRI data were collected with eyes closed.

#### MRI data pre-processing

Data were pre-processed and analyzed using FMRIB's Software Library (FSL 6.0.4) and SPM12 (Neuroimaging, 2020) and CONN Functional Connectivity Toolbox (NITRC, 2020)

All T1-weighted images were corrected for field bias using N4BiasFieldCorrection, then were segmented into grey matter, white matter, and cerebrospinal fluid using the SPM-unified segmentation pipeline. The SPM-DARTEL pipeline was used to generate a study-specific template and normalize structural data to the template using field warp and grey matter deformation maps that can be used to normalize the structural and fMRI data into common space.

The rsfMRI scans were corrected for slice timing (FSL-slice timer) and head motion (FSL-mcflirt). Any fMRI datasets with head displacement > 3mm were excluded (Hearne et al., 2017), and fMRI scans with frame-to-frame displacements > 0.40mm were censored (Power et al., 2014) with any dataset with < 85% of volumes remaining were excluded (Hearne et al., 2017). None of the datasets were excluded from the analysis because of the motion as the average motion across participants was 1.58 ± 0.75 mm. The rsfMRI data was skull-stripped (FSL-bet), resampled to isotropic resolution of 2 mm, and co-registered linearly to corresponding T1 scans in the native space (FSL-flirt). The study-specific template was linearly registered to the Montreal Neurological Institute (MNI) space, then all the individual fMRI data were spatially normalised to the MNI space (2 mm^3^ voxels). Following normalization, the fMRI data were smoothed using a 6mm Gaussian kernel of full width at half maximum.

To reduce other possible noise sources, the normalised fMRI data were further cleaned using CONN functional connectivity toolbox denoising pipeline (Whitfield-Gabrieli & Nieto-Castanon, 2012). The ART-based motion scrubbing and outlier volume removal was performed, and the motion ART artifacts were regressed out. In addition, the physiological fluctuations were removed using aCompCor algorithm (Behzadi et al., 2007) to regress out white matter and cerebrospinal fluid signals. This is achieved by using principal component analysis (PCA) of the multivariate BOLD signal within the white matter and cerebrospinal fluid masks to estimate the first five orthogonal time series to regress out the effect of the physiological fluctuations. However, the global signal was not regressed out due to its controversy as a pre-processing step (Murphy & Fox, 2017). Finally, the data were temporally filtered with band-pass (f = 0.007 – 0.1 Hz).

#### Independent component analysis

To estimate the different functional networks, a dual-regression approach was utilized to calculate temporally-concatenated probabilistic independent components (Beckmann et al., 2009), using the Multivariate Exploratory Linear Decomposition into Independent Components (MELODIC), with number of components limited to 30. Functional networks were identified through visual inspection of the ICA components based on known networks (Beckmann et al., 2005; Yeo et al., 2011), including anterior and posterior default mode network (DMN), medial-inferior temporal component of DMN, salience network (SLN) , and fronto-parietal network (FPN).

To determine if changes in behaviour correlated with changes in functional connectivity, we performed ordinary least squares regression between the standardized changes w.r.t the pre-intervention. The standardized change was calculated using the relation $\left(\Delta x-\overline{x}\right)/{x}_{\mathrm{std}}$ where *x* was the change in functional connectivity or behavioural measure given by Δ*x* = *x*_post_ − *x*_pre_, and $\overline{\boldsymbol{x}}$ and ***x***_**std**_ were the mean and standard deviation at pre-intervention.

### Statistical analyses

#### Behavioral analyses

Shapiro-Wilk tests determined assumptions of normality were violated. Wilcoxon signed–rank tests were used to compare behavioral data (SCAS, CPT 3) at pre- and post- intervention for: (i) change in anxiety scores (SCAS total and sub-total scores); and (ii) change in attention scores (CPT3 detectability, commissions, omissions and hit response time). All statistical tests were conducted using SPSS (Corp, 2019). Statistical significance was set at p<.05. (Tables 1 and 2)

**Table 1:** CALM study demographic data

Variable	Value
Participants	47
Age 9-11 years	M 10.47 years SD 0.86
Gender	
Males Females Other	31 (66%) 16 (34%) n/a
Ethnicity	
Caucasian Asian Aboriginal & Torres Strait Islander	44 (94%) 2 (4%) 1 (2%)
School type	
State Private Other (home-school)	22 (47%) 24 (51%) 1 (2%)
Handedness	
Right-handed Left-handed Ambidextrous	32 (68%) 4 (9%) 11 (23%)

**Table 2:** Wilcoxon signed rank tests and effect sizes for pre- and post- mindfulness intervention behavioural scores (anxiety and attention)

Variable	*N*	Pre-median	Post- median	Effect size r^^^	Wilcoxon (z)	P value^#^
*Anxiety*						
Total score Separation Anxiety Obsessive- Compulsive GAD Social Phobia Panic and agoraphobia Physical injury fears	42	32 4 7 7 5 4 3	27 3 6 6 5 4 3	0.4 0.6 0.6 0.1 0.2 0.1 0.1	-2.2 -3.0 -3.0 -0.5 -1.3 -0.8 -0.8	.015 .001 .001 .294 .095 .021 .224
*Attention*						
Commissions Omissions HRT Detectability	42	47 -2 492 -2	34 -2 494 -2	0.5 0.0 0.2 0.2	-2.7 -0.1 -1.6 -1.6	.003 .478 .053 .053

^^^effect size calculated as r =$\frac{z}{\sqrt{N}}$

^#^one-tailed

#### Functional connectivity (FC) analyses

To explicate the effect of the mindfulness intervention on FC, voxel-based analysis of the different brain networks was performed to estimate differences between pre- and post-intervention FC. Differences were estimated by entering individual maps into a general linear model; paired t-tests were performed with permutation testing (FSL-randomize; 5000 permutations) (Nichols & Holmes, 2002). All statistical analyses were corrected for age and gender. Results were corrected for multiple comparisons with family-wise error (p ≤ 0.05, cluster > 100 voxels) (Nichols & Holmes, 2002). In addition, we performed correlations between FC maps and cognitive variables SCAS total, detectability commissions and hit response time at pre- and post-intervention time-points (significance at p ≤ 0.05). These results were also corrected for multiple comparisons using family-wise error. We also performed correlations between FC maps and significant cognitive variables separation anxiety, hit response time and detectability to determine the change in scores (Tables 3 and 4). The change in scores was Z-scored against the mean and SD of the pre-intervention scores. These results were also corrected for multiple comparisons using family-wise error (significance at p≤ 0.05).

**Table 3:** Wilcoxon signed rank tests and effect sizes for correlational analysis for pre and post mindfulness intervention behavioural scores (anxiety and attention) *N*=38

Variable	Pre-intervention median	Post-intervention median	Effect size r*	Wilcoxon standardised test statistic (z)	P value**
*Anxiety*					
Total score Separation	32 4	27 3	0.2 0.33	-1.7 -2.9	.044 .002
Anxiety					
Obsessive- Compulsive	7	6	0.30	-2.6	.004
*Attention*					
Commissions DPR HRT	47.9 -2.05 487.64	34.0 -1.59 570.63	0.33 0.19 0.21	-2.8 -1.7 -1.83	.002 .045 .034

*effect size calculated as r =$\frac{z}{\sqrt{N}}$

**one-tailed

**Table 4:** Change Score Correlations

Network, roi and measure	beta	p-value	r2	Correlation finding
rFPN_STG Left_SC1_SEP	-0.55	0.024	0.13	Increased connectivity between right FPN to left STG was associated with a decrease in SC1_SEP
DMN_Caudate_Head_HRT	-0.98	0.028	0.13	Increased connectivity between DMN to caudate head was associated with a decrease in HRT
lFPN_1PFThal left_DPR	0.61	0.023	0.14	Increased connectivity between left FPN to left thalamus was associated with an increase in DPR
lFPN_PCC Right_SC1_SEP	0.4	0.043	0.11	Increased connectivity between left FPN to right PCC was associated with an increase in SC1_SEP

**Abbreviations for behavioural codes;**

SC1_SP – Separation anxiety (anxiety sub-scale)

HRT – Hit reaction time (attention variable)

DPR – Detectability (attention variable)

## Results

### Behavioral results

For the behavioral data, *N*=4 participants were excluded due to having an outlier score (≥ 3SD) at either time-point, hence, the final sample analyzed was *N*=42 (M 10.24yrs *SD* 1.0; males *N*=28 and females *N*=14). Participants had different ethnicities including Caucasian 94%, Asian 4%, Aboriginal and Torres Strait Islander 2%. In addition, participants were equally diverse in terms of their school type with 47% of the participants attending a state school, 51% attending a private school and 1% home-schooled. For more information about demographics, see Table 1.

#### Impact of mindfulness on anxiety (SCAS scores)

Results showed a significant difference between median pre- and post- intervention scores for total anxiety score, separation anxiety score and obsessive-compulsive anxiety score (Table 1). There was no significant difference in pre- and post- intervention scores for the variables generalized anxiety disorder, social phobia, panic, agoraphobia or physical injury fears.

#### Impact of mindfulness on attention (CPT 3 scores)

Participants performed better after the intervention, with results indicating a significant decrease in commission errors from pre- to post- intervention time-points (Table 1). There was no significant difference in pre- to post-intervention scores for the variables DPR, omissions or HRT.

### Neuroimaging results

Five participants were excluded from the rsfMRI analysis due to incomplete data acquisition resulting from image distortion artefact, thus *N=42* participants completed scans at both pre- and post-intervention time-points. Post-intervention, the group showed changes in FC at several networks including the SLN, FPN and the Medial-Inferior Temporal component (MTG) of the DMN and were corrected for multiple comparisons (p≤ 0.05). However, no significant differences were observed in anterior or posterior components of the DMN.

**Fig. 1.**
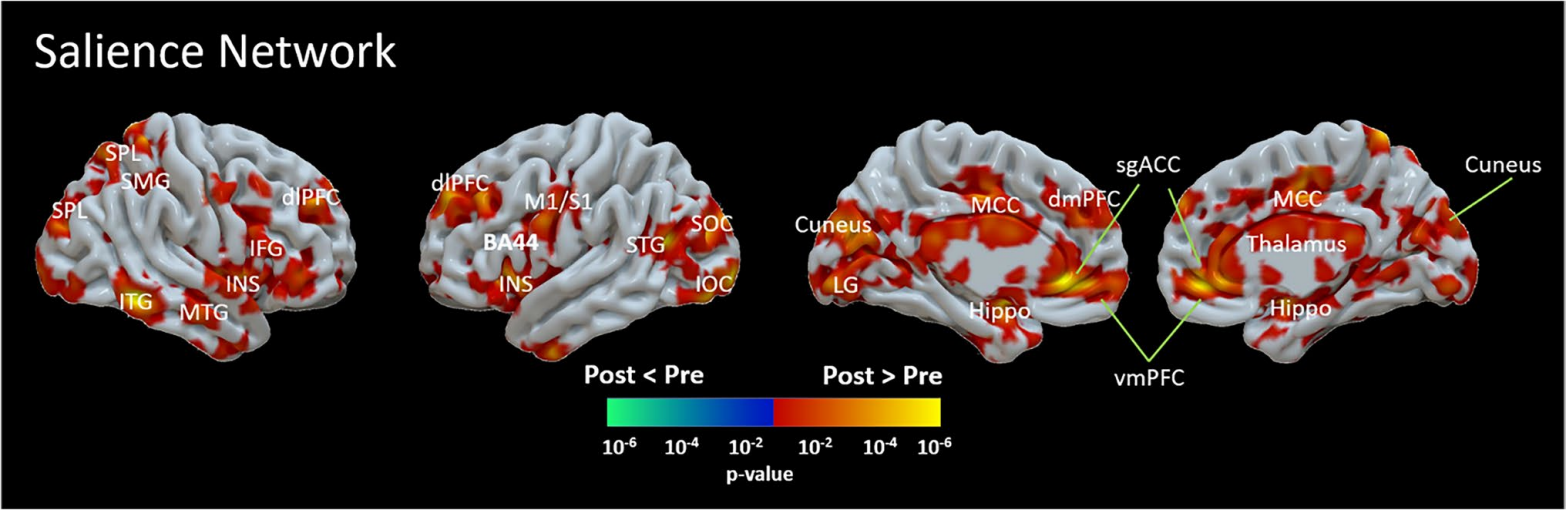
Differences between the pre- and post-intervention in functional connectivity (FC) of the salience network (SLN). The results showed increased FC of the SLN following the 10 week mindfulness intervention. The regions with increased FC of the SLN are linked to the default mode network (supra-marginal gyrus, ventromedial pre-frontal cortex -vmPFC, posterior cingulate cortex - PCC, amygdala and hippocampus, inferior temporal gyrus-ITG), visual perception (superior occipital gyrus, inferior occipital gyrus ), sensorimotor function (putamen, primary motor cortex and somatosensory cortex), cognitive functions (dorsolateral pre-frontal cortex - dlPFC, inferior frontal gyrus - IFG, superior parietal lobule), or attention (anterior cingulate cortex - ACC, mid-cingulate cortex and insula)

For the SLN, compared to pre-intervention, there was increased FC post-intervention in multiple locations, as shown in Fig. 1.

**Fig. 2.**
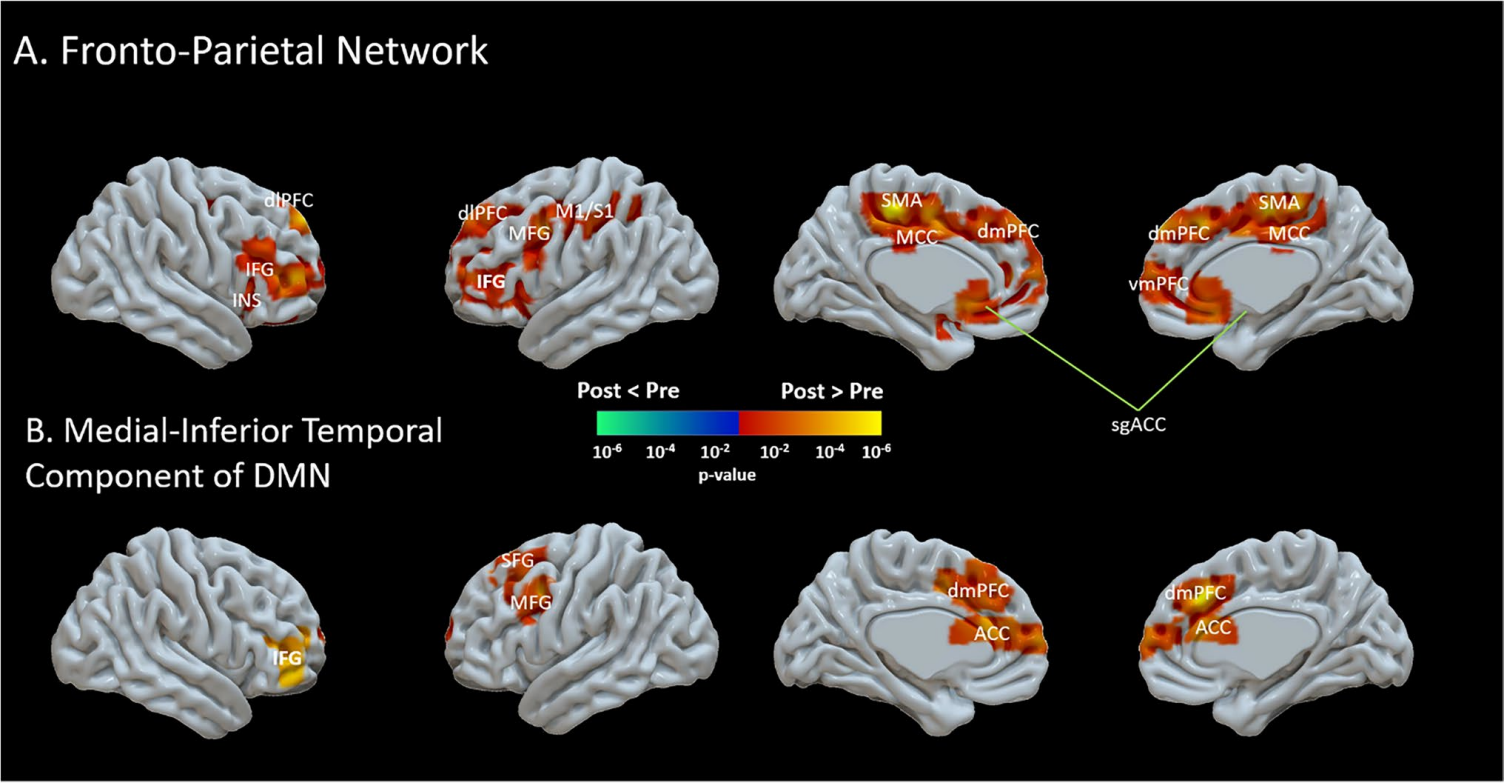
Differences between the pre- and post-intervention time-points in the functional connectivity (FC) of: (**A**) Frontal-Parietal network (FPN) and (**B**) the Medial-inferior temporal component (MTG) of the Default Mode Network (DMN). The results showed increased FC of the FPN and MTG following the 10-week mindfulness intervention. The regions with increased FC of the FPN are linked to the sensorimotor (primary sensory and motor cortices (S1, M1), supplementary motor area (SMA), middle frontal gyrus (MFG), cognitive and executive (dlPFC, dmPFC, vmPFC, inferior frontal gyrus (IFG)), and attention (insular cortex (INS), ACC). The regions with increased FC of the MTG of the DMN are linked to different brain functions including the dmPFC, ACC, IFG, MFG, superior frontal gyrus (SFG).

Similarly, the FPN showed increased FC post-intervention in the dlPFC, dmPFC, vmPFC, insula, middle frontal gyrus (MFG), inferior frontal gyrus (IFG), middle cingulate cortex (MCC), primary sensory and motor cortices and supplementary motor area as shown in Fig. 2A. Similarly, the MTG of the DMN showed increased FC post-intervention in the dmPFC, ACC, IFG, MFG and superior frontal gyrus (SFG) as shown in Fig. 2B.

### Correlational analysis

There were *N*=38 individuals with complete behavioral and MRI data at both time-points, hence, correlational analyses were conducted at the pre-intervention time-point. The HRT had a positive correlation with right FPN connectivity to left amygdala (*df* = 36, *r* = 0.73; *p* < 0.001), right hippocampus (*df* = 36, *r* = 0.47; *p* < 0.001), caudate head (*df* = 36, *r* = 0.48; *p* ≤ 0.05), left superior temporal gyrus (*df* = 36, *r* = 0.49; *p* ≤ 0.05), and precuneus (*df* = 36, *r* = 0.53; *p* ≤ 0.05). In addition, SCAS total score was positively correlated with left FPN connectivity to right caudate head (*df* = 36, *r* = 0.52; *p* ≤ 0.05). Furthermore, the posterior DMN connectivity to right PCC (*df* = 36, *r* = 0.61; *p* ≤ 0.05) and left PCC (*df* =36, r = 0.52, p ≤0.05), and precuneus (*df* = 36, *r* = 0.49; *p* ≤ 0.05) were positively correlated with the attention variable HRT. No correlations were observed between the behavioral measures and the salience FC. In contrast to the pre-intervention time-point, there were no significant correlations at the post-intervention time-point for any of the functional networks.

Additionally, we investigated the correlation between the change in the functional connectivity and the change in the behavioral outcomes at the regions where we observed functional connectivity changes. The results showed improved behavioral measures are correlated with the functional connectivity increases as shown in Table 4.

## Discussion

This is the first study to investigate effects of a mindfulness intervention on FC in resting-state networks in a preadolescent cohort with sub-clinical anxiety-related attention impairments. Understanding mindfulness-induced neural and associated behavioral changes is crucial to support the efficacy of early interventions (Sylvester et al., 2012). Our results showed increased FC in the resting-state networks investigated [SLN, FPN, and DMN (MTG)] following the mindfulness intervention. Increased FC was observed in the SLN and FPN, suggesting improvements in cognitive control and emotion regulation. However, contrary to what was observed in the literature in adults showing reduced DMN functional connectivity (Brewer et al., 2011; Hasenkamp et al., 2012; Marusak et al., 2018), our study showed increased functional connectivity in the DMN in a pre-adolescent population suggesting possible functional connectivity changes were associated with development of the DMN in the study population. These observed changes in FC were further associated with improved behavioral outcomes post-intervention.

The SLN, including key nodes insula and ACC (Uddin, 2015), is involved in the detection, filtering and orientation to salient stimuli (Menon & Uddin, 2010). Decreased FC in the SLN to the prefrontal regions and insula has been implicated in deficits in emotion regulation and cognitive control, processes recognized as underlying anxiety and attention disorders (Etkin et al., 2011; Uddin et al., 2011). Specifically, the insula, an attention processing hub, plays an important role in interoceptive awareness, emotional responses and high level cognitive control (Craig, 2003; Menon & Uddin, 2010). Whilst the prefrontal regions are involved in the mediation of higher order cognitive processes such as rational thinking and goal planning essential for cognitive control and affect regulation (Dumontheil, 2014; Fuster, 2002). Therefore decreased FC in the SLN to these regions will reduce salience processing of internal and external stimuli, critical to support cognitive control and emotion regulation (Uddin, 2015).

Recent research has associated mindfulness interventions with increased FC in the SLN to the insula, vmPFC, dmPFC, dlPFC, ACC, hippocampus and amygdala and improvements in anxiety symptoms (Marusak et al., 2018; Tang et al., 2015), consistent with our findings. Specifically, increased FC in the prefrontal regions has been associated with improved cognitive control required for effective processing of emotionally salient stimuli (Etkin et al., 2011; Kober et al., 2008). Furthermore, our observation of increased SLN connectivity to the dlPFC, hippocampus and amygdala has been associated with improvements in switching attention from emotionally salient events. (Forster et al., 2015; Zheng et al., 2017).

The FPN is the central executive network, and with frontal regions, supports executive control of attention (Corbetta & Shulman, 2002; Petersen & Posner, 2012). Decreased FC in the FPN is associated with deficits in executive function, specifically cognitive control and emotion regulation and has been reported in children with impairments in attention and anxiety (Rosenberg et al., 2016). Mindfulness training has been associated with increased FC in the FPN and increased cognitive control and emotion regulation in adult and youth populations (Hölzel et al., 2013; Vago & David, 2012). Our findings indicate increased FC to the vmPFC, dlPFC, vmPFC, IFG, MFG, and insula in our cohort. The insula has been implicated in introspective thoughts (Critchley et al., 2004), thus increased FC to the dlPFC and vmPFC may modulate attention to anxious thoughts (Menon & Uddin, 2010; Namkung et al., 2017). Moreover, an overall decrease in participants’ anxiety scores was supported by improved FC in these regions. Increased FC to the IFG and MFG has been implicated in increased response inhibition to irrelevant internal emotional stimuli, thus supporting sustained attention (Japee et al., 2015; Petersen & Posner, 2012). Furthermore, an overall decrease in participants’ commission scores supports improvement in inhibitory control.

Attention to self-referential thoughts or ‘mind-wandering’ has been associated with increased activation in the DMN during rsfMRI, as well as anxiety and attention issues (Brewer et al., 2011; Marusak et al., 2018). Mindfulness training has been shown to decrease activation in the DMN in adults, thus decreasing mind-wandering (Jha et al., 2007). Our result of increased FC within the DMN regions of dmPFC, ACC, IFG, MFG, and SFG may underpin improvements in emotion regulation and corroborate recent findings by (Hafeman et al., 2020). Previous studies established that developmental FC changes in children are characterized by weakened short-range FC that strengthens to long-range FC in adulthood (Kelly et al., 2009; Uddin et al., 2010). Our findings of increased FC were observed after a relatively short period of 10-12 weeks between MRI scans. Taken together, we suggest that FC changes within networks in this study are due to the mindfulness intervention, and less likely associated with developmental changes. Furthermore, increased FC between the SLN, FPN and DMN following the mindfulness intervention may indicate improved cognitive control and emotion regulation (Marusak et al., 2018). The SLN plays a crucial role in switching activation between the FPN and DMN, as these networks support processing the emotional salience of irrelevant stimuli and are often dysfunctional in children with anxiety-related attention impairments (Sha et al., 2019).

Positive correlations were identified pre-intervention in the FPN to several brain regions for the HRT and the SCAS total and in the DMN to several brain regions for the HRT, however not post-intervention. The pre-intervention, the right FPN was correlated with slower response times, often associated with inattentiveness. Specifically, increased FC to the left amygdala and right hippocampus sends direct projections to the caudate, negatively affecting goal-directed behavior (Grahn et al., 2008). The FPN connectivity to the caudate showed a positive correlation with SCAS total score at pre-intervention, indicating increased FC is associated with elevated anxiety symptoms observed in this study. Correspondingly, greater caudate activation has been reported in children with high levels of behavioral inhibition, considered a risk factor for anxiety (Lahat et al., 2018; Vincent et al., 2008). However, this response was improved in our participants post the mindfulness intervention, with no significant correlations observed between the FPN FC and the HRT or total SCAS score, suggesting normalization of participants’ FC.

Momentary lapses of attention have been associated with increased activity in the DMN wherein strong interactivity of key nodes PCC/precuneus, may be linked to slower HRT (Cavanna & Trimble, 2006; Weissman et al., 2006). Moreover, the correlation of DMN connectivity with HRT pre-intervention might arise from increased self-awareness and reduced task performance, which is known to take place in participants with behavioral deficits (Alexopoulos et al., 2012; Zhang & Li, 2012). At post-intervention, these observed correlations of the DMN connectivity were not significant, suggesting the mindfulness intervention may have improved FC and behavioral performance (Brewer et al., 2011). Taken together, the lack of correlations post-intervention in the FPN and DMN networks suggests the mindfulness intervention altered FC in these networks and reduced behavioral scores. Thus, the observed pre-intervention correlations and the behavioral variables may be markers for anxiety-related attention impairments in pre-adolescent populations.

Furthermore, the functional connectivity changes between post- and pre-intervention in different networks were correlated with the behavioral improvements as a result of the mindfulness intervention. For instance, the increased connectivity between right FPN to left STG was correlated with the decrease in separation anxiety, suggesting its role in the improvement of emotion regulation contributed to a decrease in separation anxiety score (Zhao et al., 2014) It is also recognised that the increased functional connectivity in the FPN network has been associated with increased cognitive control in healthy populations (Zanto & Gazzaley, 2013), and reduced severity of separation anxiety symptomatology (Sylvester et al., 2012). In addition, STG functional connectivity was suggested to be associated with dysfunctional emotion regulation due to its role in social recognition and perception of emotions as part of the mirror neuron circuit (Kohn et al., 2014). Similarly, the DMN connectivity to caudate head was increased in correlation with a decrease in Hit Reaction Time, suggesting improved performance in association with improved self-perception and improvement of emotion regulation and cognitive performance. DMN has been often associated with off task internal mentation or with goal-oriented tasks that require self-referential processing, with recent reports suggesting its involvement in higher cognition (Vatansever et al., 2018). Such a shift in the functional connectivity within the DMN might suggest an alternative pathway for the information processing resulting in reduced reaction time (Vatansever et al., 2015).

Moreover, the FPN connectivity increase to the PCC was also correlated with the increase in recovery in separation anxiety, suggesting that the increased FPN connectivity to the DMN hubs (namely PCC) are to be playing a role in the recovery of anxiety levels towards normal levels. This is also supported by the previous research suggesting the involvement of the PCC in different functions including emotion regulation, attention control (Fox et al., 2005), and the failure to control the DMN activity might interfere with emotion control and regulation in patients with anxiety disorders (Pletzer et al., 2015; Weissman et al., 2006; Sylvester et al., 2012).

However, the increased FPN to thalamus connectivity was correlated with the increase in the attention variable detectability which means increased connectivity was observed with reduced performance. This is the opposite to what we expected to see of increased functional connectivity in association with better performance in attention (Hart et al., 2013). However, it is also important that this was not contrasted against controls, suggesting that these participants with attention problems linked to their anxiety require further investigations to validate this.

Our study has several limitations that require mentioning. Firstly, this is not a randomised control study as a control group was not included in the design, as sub-clinical anxiety is pervasive in preadolescent children, with approximately up to 70% of children reporting they worry “every now and then” (Cartwright-Hatton, 2006; Muris et al., 2002). Secondly a mindfulness scale was not included, as the concepts of metacognition and interoceptive awareness are still developing during pre-adolescence; thus, researcher involvement to clarify questions may have increased risk of response bias. Thirdly, we did not collect the social economic status of the participants or their families, which might contribute to the outcome changes in the current study, however, it is important to note that there was an equal distribution between private and public schools in the current study. Future studies with more comprehensive demographic and behavioural measures would be able to address this concern. Finally, as this was a group program, some improvements may be attributed to social interactions and group discussions.

## Conclusions

Our study has shown that mindfulness was associated with improved anxiety and attention behavioural scores as well as altered FC in several interconnected resting-state networks. The mindfulness induced behavioural and FC findings should be considered in the context of developmental changes occuring in pre-adolescent children. Furthemore, the findings of positive correlations at pre-intervention in the FPN and DMN networks to several brain regions with the attention variable HRT, and the anxiety variable SCAS total score, suggests that excessive FC in these networks may be a marker of anxiety-related attention impairments in sub-clinical populations. Our findings suggest mindfulness strategies may be an effective intervention for sub-clinical anxiety-related attention impairments in pre-adolescent children, however need to be considered in the context of developing functional connectivity.
